# An *in vitro* study on the efficacy of removing calcium hydroxide from curved root canal systems in root canal therapy

**DOI:** 10.1038/ijos.2017.14

**Published:** 2017-06-23

**Authors:** Ying Wang, Li-Yang Guo, Hong-Zhi Fang, Wen-Ling Zou, Ying-Ming Yang, Yuan Gao, Hui Yang, Tao Hu

**Affiliations:** 1 State Key Laboratory of Oral Disease, National Clinical Research Center for Oral Diseases, West China Hospital of Stomatology, Sichuan University, Chengdu, China; 2 Department of Endodontics, The Affiliated Stomatology Hospital, School of Medicine, Zhejiang University, Hangzhou, China; 3 Department of Stomatology, The Third People’s Hospital of Chengdu, Chengdu, China; 4 State Key Laboratory of Oral Disease, National Clinical Research Center for Oral Diseases, Department of Preventive Dentistry, West China Hospital of Stomatology, Sichuan University, Chengdu, China; 5 State Key Laboratory of Oral Disease, National Clinical Research Center for Oral Diseases, Department of General Dentistry, West China Hospital of Stomatology, Sichuan University, Chengdu, China

**Keywords:** calcium hydroxide, curved root canal system, irrigation, removal

## Abstract

To compare the efficacy of various irrigants (citric acid, ethylenediaminetetraacetic acid (EDTA) and NaOCl) and techniques in removing Ca(OH)_2_ in two types of curved root canal systems, simulated root canals with specific curvatures were used to investigate the effects of different irrigants and instruments on Ca(OH)_2_ removal. The optimal methods were verified on extracted human teeth. Simulated root canals were assigned to one of two groups based on the irrigation solution: 10% citric acid or 2.5% NaOCl. Each group was divided into four subgroups according to the technique used to remove Ca(OH)_2_. The percentage of Ca(OH)_2_ removal in different sections of root canals was calculated. On the basis of the results obtained for the simulated canals, 10% citric acid and 17% EDTA were applied to remove Ca(OH)_2_ from the extracted human teeth with curved root canal systems. The teeth were scanned by micro computed tomography to calculate the percentage of Ca(OH)_2_ removal in the canals. In simulated root canals, we found that 10% citric acid removed more Ca(OH)_2_ than 2.5% NaOCl in the 0–1 mm group from the apex level (*P*<0.05). Ultrasonic and EndoActivator activation significantly removed more Ca(OH)_2_ than a size 30 K file in the apical third (*P*<0.05). However, there were no significant differences in any sections of the canals for 10% citric acid or 17% EDTA in removing Ca(OH)_2_ in extracted human teeth. We concluded that it was effective to remove residual Ca(OH)_2_ using the decalcifying solution with EndoActivator or Passive Ultrasonic Irrigation in a curved root canal system. A protocol for Ca(OH)_2_ removal was provided based on the conclusions of this study and the methods recommended in previous studies.

## Introduction

Overwhelming evidence indicates that microorganisms have a fundamental role in the pathogenesis of periradicular diseases.^1^ To eliminate the remaining microbes after root canal preparation and to prevent an interappointment reinfection of the root canal system, intracanal medications are recommended.^2^ Calcium hydroxide [Ca(OH)_2_] has been widely used as an intracanal medication owing to its good antibacterial properties and biocompatibility.^2^ However, some studies have indicated that Ca(OH)_2_ inactivates endotoxin^3^ and impedes the increase in cytokine chemical inflammatory mediators^4, 5^ to inhibit periapical inflammation after a root canal cleaning procedure.

The antibacterial effect of Ca(OH)_2_ is most effective after direct contact with microorganisms.^2, 6^ Therefore, it is important to obtain a compact and homogenous filling of Ca(OH)_2_ in the entire root canal system.^6^ Before obturation, Ca(OH)_2_ should be completely removed from the root canal system to avoid any possible negative influence on treatment. Residual Ca(OH)_2_ might lead to apical leakage^7^ and reduced sealer adaptation,^8^ and might interact with zinc oxide eugenol^9^ and decrease the dentin bond strength.^10^ Therefore, any Ca(OH)_2_ that is placed in the root canal system as an intracanal medication must be completely removed before root canal obturation.

The most frequently described method for removing Ca(OH)_2_ from the root canal is the instrumentation of the root canal with the master apical file in combination with copious irrigation.^2, 11^ Previous studies have investigated the efficacy of Ca(OH)_2_ removal with different irrigants, equipment and techniques. Rödig *et al.*^12^ indicated that decalcifying solution, such as citric acid and ethylenediaminetetraacetic acid (EDTA), was significantly more effective than sodium hypochlorite and water. Decalcifying solutions in endodontics comprise chelators and acid.^13^ Rotary instruments, the self-adjusting file system and high-efficiency irrigation systems, such as passive ultrasonic irrigation and the EndoVac system, were significantly more effective than conventional syringes.^14, 15, 16^ However, none of these methods completely removed the Ca(OH)_2_ intracanal medication. Although more techniques have been proposed to remove Ca(OH)_2_, an effective and systematic Ca(OH)_2_ removal strategy is not available.

Most studies of Ca(OH)_2_ removal have used extracted human teeth with straight root canals.^12^ Some researchers have established artificial standardized grooves in root canal apices to study Ca(OH)_2_ removal for irregular parts of the root canal system.^14, 15, 16^ To evaluate the quantity of Ca(OH)_2_ remaining after removal with various techniques in curved root canal systems, extracted human teeth with curved root canals with a certain range of curvature were selected in previous studies.^17^ Because canal anatomy is one of the main confounding factors affecting the experimental results,^18^ especially in complicated root canal systems, the experimental groups should be anatomically comparable to maintain a consistent baseline for the validity of experimental results. It is difficult for extracted human teeth to meet this condition as experimental specimens because of their heterogeneity. To research the shaping ability of rotary instruments in severely curved canals, Schäfer attempted to ensure standardization of the experimental groups by balancing them with respect to the root canal angle and the radius of canal curvature. The homogeneity of the groups was examined using statistical methods.^19^ Simulated root canal models were proposed to create an undifferentiated experimental group in studies regarding a certain curvature. Some researchers used simulated root canals to mimic the actual curved canal system.^20^ This *in vitro* model ensures standardization, provides a consistent baseline for each experimental group and avoids the confounding factors from root canal anatomy, such as the isthmus, length, curvature and radius. The curved root canal models used in previous studies offer a new choice to establish a more rational model for Ca(OH)_2_ removal research in curved root canal systems.

The objective of the present study was to compare the efficacy of different irrigants (citric acid, EDTA and NaOCl) and techniques (rotary instrumentation, sonic and passive ultrasonic irrigation) in removing Ca(OH)_2_ in two curved root canal models: simulated root canals and extracted human teeth.

## Materials and methods

### Sample preparation for simulated curved root canals

This experiment consisted of 20 simulated curved root canals (Endo Training Block; DentsplyMaillefer, Ballaigues, Switzerland) that were 16.5 mm long. The degree of curvature was 45.2°, and the radius of canal curvature was 6.1 mm, according to Schneider’s method.^21^ The canals were prepared to F3 (apical size 30 with a 0.09 taper) by the same operator using a ProTaperNiTi Rotary System (DentsplyMaillefer, Ballaigues, Switzerland) according to the manufacturer’s instructions.^22^ 5 mL of 2.5% NaOCl was used after each instrument, and 5 mL of distilled water was used as the final rinse. The simulated root canals were dried with paper points. The apical foramen was then filled with wax from the outside.^22^ Canals were filled with Ca(OH)_2_ paste (UltracalXSOptident, International) using a 30-gauge NaviTip (Ultradent products Inc., South Jordan, UT, USA) starting from the apical aspect with the needle slowly advancing coronally until the paste was visualized at the canal orifice.^6^ The access cavities were sealed with a cotton pellet and temporary filling material (GC corporation, Tokyo, Japan). The root canals were stored at 37 °C and 100% relative humidity for 7 days.

### Different irrigants and different instruments in calcium hydroxide removal in simulated curved root canals

**Figure 1:**
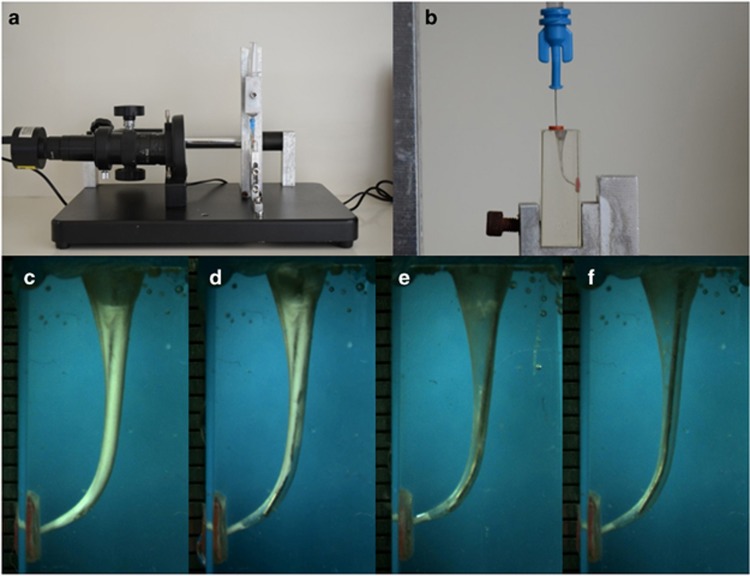
**The experimental apparatus and operational process of Ca(OH)_2_ removal.** (**a**) The experimental set-up, including a camcorder and fixing device. (**b**) Simulated curved root canals and syringe needle. (**c**–**f**) Images after 0 mL, 5 mL, 10 mL and 15 mL of irrigation. Syringe needle inserted 2 mm from the apex. Between each 5-mL irrigation, different instruments were applied to remove Ca(OH)_2_.

Before the experiment, the temporary filling material was removed, and the Ca(OH)_2_ in the simulated canals was loosened using a 15 K file. There were two experimental groups based on the irrigation solutions used to remove Ca(OH)_2_. In group 1, the canals were irrigated with 10% citric acid. In group 2, the canals were irrigated with 2.5% NaOCl. All canals were irrigated following a standardized protocol. The operating cycle included 5 mL of irrigant and different techniques, and the final rinse was performed after two operating cycles. A 30-gauge NaviTip (Ultradent) needle was used at a length that was 2 mm shorter than working length,^17^ and the pressure applied to the plunger was standardized to 19.6 N using the device designed for this experiment (Figure 1a and 1b). Each group was divided into four subgroups according to the technique used to remove Ca(OH)_2_. In subgroup 1(*n*=10), canals were filed manually with a 30 K file in a circumferential filing action at working length for 10 s. In subgroup 2 (*n*=10), canals were filed with a size F3 instrument with a rotating-lifting action at working length for 10 s. In subgroup 3 (*n*=10), ultrasonic activation was delivered for 30 s with a stainless steel ultrasonic file 15/0.2 (Irri-Safe Satelec; Acteon Group, Merignac, France) mounted on a Suprasson P5 Booster ultrasonic unit (Satelec, Acteon Group, Merignac, France) with the power setting at 5.^17^ In subgroup 4 (*n*=10), sonic activation was delivered for 30 s between each irrigant using the EndoActivator (Advanced Endodontics, Santa Barbara, CA, USA) set at 10 000 cycles per minute with a 35/0.04 tip according to the manufacturer’s instructions. The final rinse was performed with 3 mL of distilled water after two cycles of irrigation. The transparent blocks were covered with adhesive tape during the instrumentation phase. After ensuring that the residues in each root canal were adequately cleaned, the simulated root canals were reused. Each canal was reused three times.

### Efficacy of calcium hydroxide removal in simulated curved root canals

The Ca(OH)_2_ removal process was recorded by a camcorder (ScopeTek DCM 310; ScopeTek, Hang Zhou, China) operated in macro mode (Figure 1a). A ring light was fixed on the camcorder to ensure the images were obtained with a standard light source. After the removal of Ca(OH)_2_, the images were obtained using a camcorder. The images were analysed using Image-Pro Plus 6.0 (Media Cybernetics, Rockville, MD, USA) on a laptop computer (HP, Palo Alto, CA, USA) with a 1 366 × 768 screen pixel resolution to quantify the proportion of pixels for residual Ca(OH)_2_ compared with the total area of the root canal wall. The software identified the residual area according to the different grey level between the residual area and the clear area. Then, the percentage of the Ca(OH)_2_ removal area was calculated. The apical third was re-divided into three portions according to their distance from the apex: 0–1 mm, 1–3 mm and 3–5 mm. The percentage of Ca(OH)_2_ removal in the apical, middle and coronary thirds together with the three portions in the apical part of the root canal was calculated. Two endodontists independently evaluated the images. When there were disagreements about the image, another endodontist was asked to perform a third evaluation to achieve a final consensus. The presence of Ca(OH)_2_ in the apical third was scored (absence was scored as “0”, and presence was scored as “1”) according to the three portions determined in the apical part.

### Sample preparation for the extracted teeth

A total of 24 recently extracted human teeth with at least one curved root and curved root canal were selected. These teeth were obtained from the Oral Surgery Department of West China Hospital of Stomatology, and this study was approved by the Ethics Committee of West China Hospital of Stomatology. The degree of curvature was measured according to Schneider’s technique with CBCT,^23^ and the radius of canal curvature was calculated. Teeth with a radius of curvature ranging from 3.5 to 10.1 mm and a degree of curvature ranging from 25.2° to 47.7° were included (Table 1). Two similar groups were established with respect to the degree and the radius of curvature. The homogeneity of the two groups was assessed using the paired *t*-test (Table 1).

The working length was established as 1 mm less than the length of the initial instrument (size 10) at the apical foramen. Root canals were prepared to F3. The preparation and Ca(OH)_2_ application procedures were the same as mentioned above. Teeth were stored at 37 °C and 100% relative humidity for 7 days.

### Different irrigants and different instruments in calcium hydroxide removal in the curved root canals of extracted teeth

The results from the simulated curved root canals indicated that decalcifying solution was more effective in removing Ca(OH)_2_. To verify the efficacy of commonly used decalcifying solution in removing Ca(OH)_2_, 10% citric acid (group 1) and 17% EDTA (group 2) were applied in the two groups. EndoActivator was used for 30 s between each 5-mL irrigation. A final rinse was performed with 2 mL of irrigant and 3 mL of distilled water after two cycles of irrigation. The total activation time was 60 s. Root canals were dried with paper points after the Ca(OH)_2_ removal procedure. Other operations followed the procedure of the simulated root canals mentioned above.

### Micro-computed tomography scanning

The teeth were scanned by micro-computed tomography (micro-CT; μCT 5.0, SCANCO Medical AG, Brüttisellen, Switzerland) with an isotropic voxel size of 38 μm at the following time points: after root canal instrumentation, after Ca(OH)_2_ application and after Ca(OH)_2_ removal. All cross-sections obtained were roughly perpendicular to the long axis of the root. The image serials were analysed using ImageJ. The volume of residual Ca(OH)_2_ was measured. Then, the percentage of Ca(OH)_2_ removal in the canals was calculated according to the method described in Ma’s research.^24^

### Statistical analysis

Data were presented as the mean±standard deviations (s.d.). Differences between the groups were determined using an unpaired Student’s *t*-test. ANOVA with *post hoc* Tukey’s test was used for statistical analysis at a 95% confidence level. Statistical significance was established at *P*-values less than 0.05.

## Results

**Figure 2:**
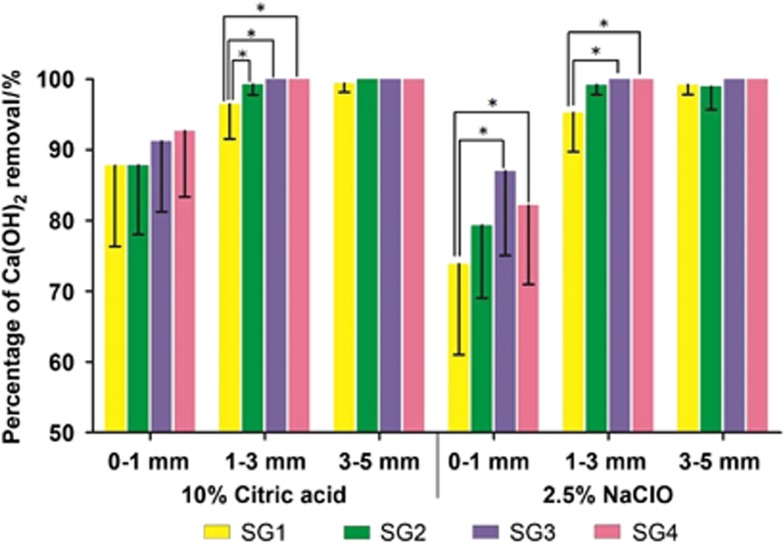
The percentage of Ca(OH)_2_ removal in each region of the apical third with 10% citric acid or 2.5% NaOCl combined with different instruments (*P<0.05).

**Figure 3:**
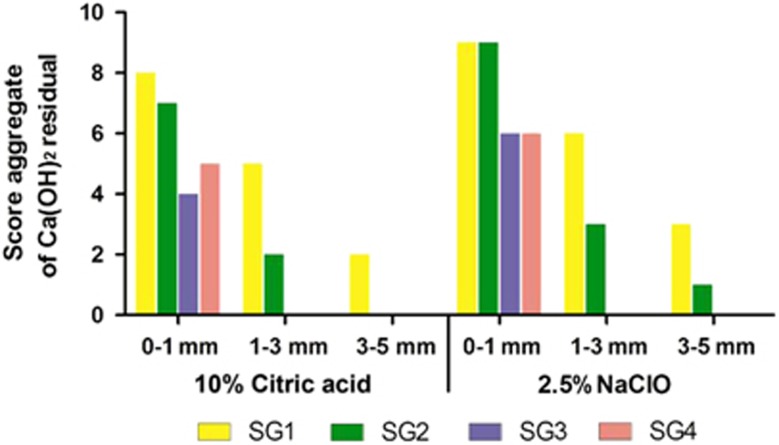
**Score distribution for the three regions in the apical third with 10% citric acid and 2.5% NaOCl.**

The percentages of Ca(OH)_2_ removal in simulated root canals are shown in Table 2 and Table 3 and Figure 2. The scores of the residual Ca(OH)_2_ are shown in Figure 3.

In each group, Ca(OH)_2_ was almost completely removed in the middle and coronal thirds. For the apical third, ultrasonic irrigation and EndoActivator removed more Ca(OH)_2_ than the size 30 K file in the 10% citric acid group, but the difference was not significant (*P*>0.05). In the 2.5% NaOCl group, the 30 K file removed less Ca(OH)_2_ than ultrasonic irrigation and the EndoActivator (*P*<0.05). There were no significant differences between the groups in the apical third (*P*>0.05).

In the apical 1–3 mm region, 10% citric acid with a 30 K file (96.48%±4.90%) was not as effective as rotary instrumentation (99.29%±1.50%), ultrasonic (100%±0%) or EndoActivator activation (100%±0% *P*<0.05). However, there were no significant differences in other root canal regions (*P*>0.05). For the 2.5% NaOCl group, ultrasonic and EndoActivator activation significantly removed more Ca(OH)_2_ than the size 30 K file in the apical one-third and the apical 0–1 mm and apical 1–3 mm groups (*P*<0.05). For the two groups, Ca(OH)_2_ was completely removed at the 1–5 mm level using ultrasonic irrigation and the EndoActivator. At the apical 0–1 mm level, the 10% citric acid group removed significantly more Ca(OH)_2_ than 2.5% NaOCl when using a 30 K file (*P*<0.05). No significant differences were noted for other subgroups at the 0–1 mm level (*P*>0.05), but the 10% citric acid group removed more Ca(OH)_2_. On the basis of the results of the scores in different segments, we found that residual Ca(OH)_2_ frequently remained in the apical 0–1 mm segment. Ultrasonic and EndoActivator activation could improve the Ca(OH)_2_ removal in the apical 1–3 mm and 3–5 mm sections.

**Figure 4:**
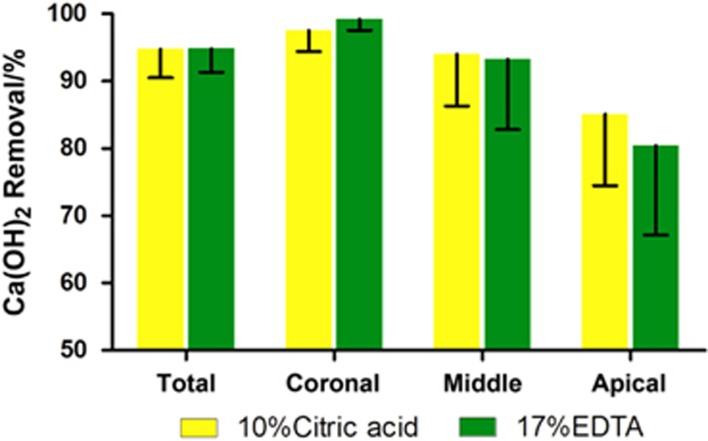
**The percentage of Ca(OH)_2_ removal for the entire canal and each third of the root canal system.**

**Figure 5:**
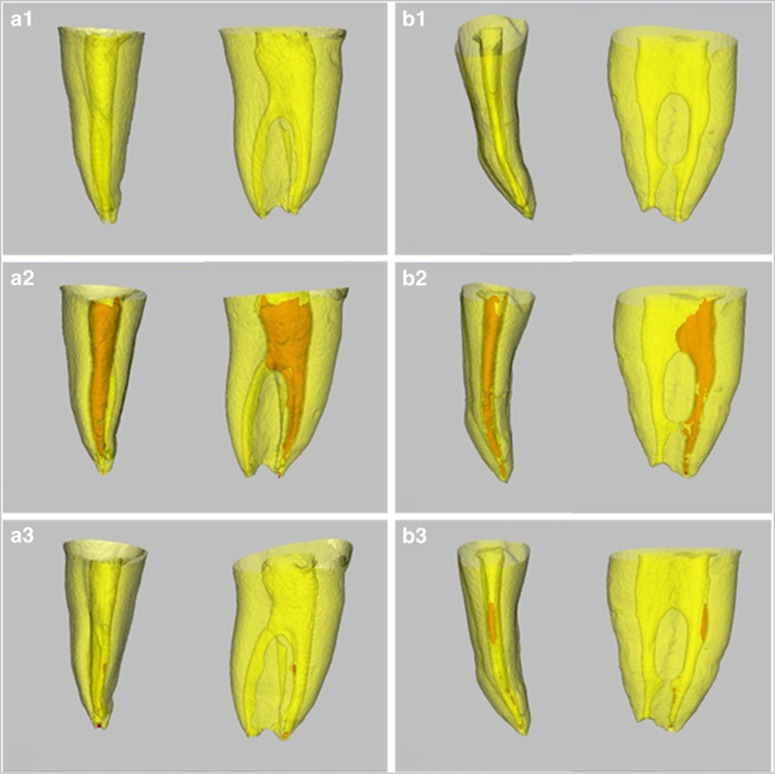
**Micro-CT reconstructed images of extracted teeth. (a1–a3)** Reconstructed images after root canal preparation, Ca(OH)_2_ medication and removal with 10% citric acid. **(b1–b3)** Reconstructed images after root canal preparation, Ca(OH)_2_ medication and removal with 17% EDTA. EDTA, ethylenediaminetetraacetic acid; micro-CT, micro-computed tomography.

In human extracted teeth, the effect of two decalcifying solutions in removing Ca(OH)_2_ is illustrated in Figure 4. There were no significant differences in any sections of the canal irrigated either with 10% citric acid or with 17% EDTA in removing Ca(OH)_2_ (*P*>0.05). For 33% of the samples, Ca(OH)_2_ was completely removed by 10% citric acid. Residual Ca(OH)_2_ was found in the apical one-third, isthmus and irregular parts (Figure 5).

## Discussion

This study investigated the effect of decalcifying solution and NaOCl on the removal of Ca(OH)_2_ in simulated curved root canals with different instruments. Ca(OH)_2_ could not be completely removed in all groups. However, decalcifying solution combined with sonic or ultrasonic agitation was the most effective method for removing Ca(OH)_2_ in curved root canal systems. Sonic or ultrasonic irrigation enhanced the agitation and shearing stress of irrigants in the root canal wall, which was effective in removing debris in root canals.^16, 25^ In a previous study, the use of citric acid as a decalcifying solution was more effective than NaOCl in Ca(OH)_2_ removal,^12^ and similar results were found in the present study. This finding might be explained by the fact that citric acid and Ca(OH)_2_ underwent a neutralization reaction. In addition, citric acid is a good chelating agent for removing the smear layer. However, NaOCl had a limited ability to dissolve inorganic substances, such as calcium.^17, 26^

Residual Ca(OH)_2_ mainly remained in the apical part of the curved root canal. Residual Ca(OH)_2_ was frequently located at the 0–1 mm region from the apex, followed by a 1–3 mm region from the apex. The result was similar to previous studies in which more Ca(OH)_2_ remained in the apical one-third.^15, 24^ Investigations that used passive ultrasonic irrigation and a master apical file to remove Ca(OH)_2_ found that there were no significant differences in the apical one-third.^15^ In this study, significant differences appeared between the 30 K file and sonic and ultrasonic irrigation groups when using 2.5% NaOCl. The findings might be explained by the differences in the experimental models and the curvature and morphology of the root canal. From there constructed images of micro-CT after Ca(OH)_2_ removal, the residual easily appeared in the apical one-third, isthmus and irregular regions. Because of the complicated anatomy of the human root canal system, residual Ca(OH)_2_ frequently remained in these regions.^16, 24, 27, 28^ Because the effectiveness of irrigation could depend on both the mechanical flashing action and the chemical ability to dissolve tissue,^29^ it may be a good choice to apply the mechanical flushing action in combination with the decalcifying solution to remove Ca(OH)_2_ in the various root canals.

There were two aspects important for choosing instruments to remove Ca(OH)_2_. First, the instruments should enhance the agitation or shearing stress of irrigants in the root canal wall, such as passive ultrasonic irrigation, EndoActivator and EndoVac.^16, 25, 28, 30^ The second aspect is mechanical removal realized by using instruments that fully contact the root canal wall.^14, 30, 31^ Moreover, some researchers have used both mechanical removal and irrigant agitation to enhance the effects of removal.^17, 32^

Determining the most appropriate and effective method for removing Ca(OH)_2_ is important. The removal procedure has the potential to alter canal wall morphology. The decalcifying solution has the ability to demineralize dentin, and it takes more than 60 s to establish a trend of saturation.^16, 26^ For EDTA, if given sufficient time to soften, a 50-μm depth is capable of decalcifying dentin.^29^ Thus, the canal diameter would increase by 0.1 mm. With a final rinse of NaOCl after the decalcifying solution, dentin erosion increases.^29^ The mechanical removal of Ca(OH)_2_ with root canal preparation instruments may affect the shape of the prepared root canal.^21^ Recently, a study showed that the file-to-wall contact during ultrasonic activation of the irrigant occurred in all case studies.^33^ Therefore, the ultrasonic technique also has the potential to alter canal morphology unpredictably during root canal irrigation when removing Ca(OH)_2_. An investigation demonstrated that mechanical root canal preparation could generate high forces, resulting in dentinal defects and even dentin fractures.^34^ It is still unknown whether the use of decalcifying solution and mechanical instruments to remove Ca(OH)_2_ could increase the risk of dentinal defects, dentin fracture or root fracture. However, theoretically, the weak dentin wall could be a contributing factor to dentin fracture and root fracture. To prevent this scenario, some details must be highlighted, such as controlling the exposure time in decalcifying solution, using irrigants at the proper concentrations and sequence, and choosing instruments that are more gentle on the canal wall.

**Figure 6:**
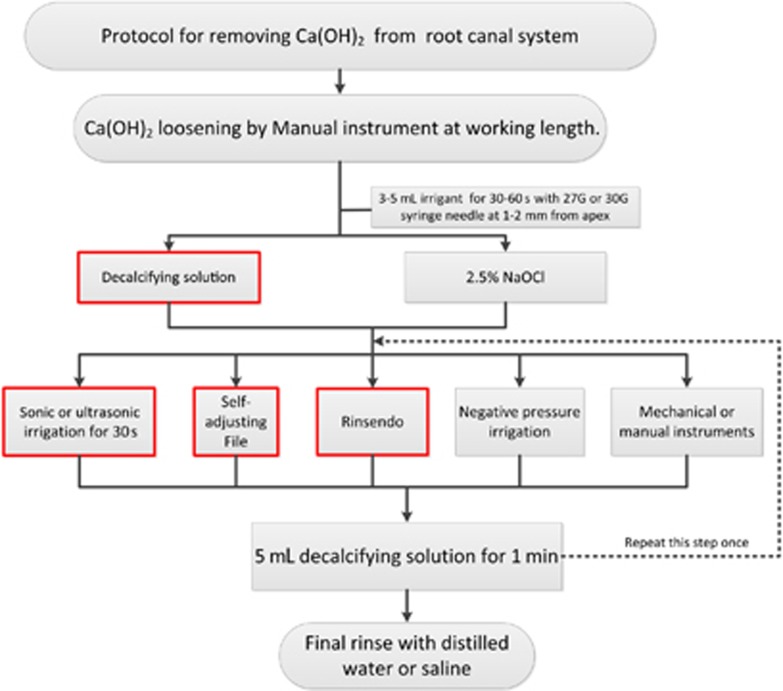
**Recommended protocol for Ca(OH)_2_ removal (the red box indicates the preferred method).**

From previous studies, we found that many factors could affect the removal efficacy of Ca(OH)_2_, including the position of the syringe needle;^35^ the type, dosage and proportion of irrigants;^11, 13^ and the application of instruments.^14, 16, 17, 24, 28^ However, no recommended Ca(OH)_2_ removal protocol is available that could be referenced in clinical practice. Therefore, on the basis of the conclusions of this study and the methods recommended in previous studies, a Ca(OH)_2_ removal protocol for curved root canals was proposed (Figure 6). Considering the various factors affecting calcium hydroxide removal, this system is a stable and effective method to remove Ca(OH)_2_ from the root canal system. This protocol uses more than 10 mL of irrigant. According to the clinical situation, the dosage of flushing fluid and times for instrument usage should be increased.

## Conclusion

In this study, Ca(OH)_2_ could not be completely removed in most of the experimental groups. The decalcifying solution with the EndoActivator or passive ultrasonic irrigation effectively removes residual Ca(OH)_2_ in a curved root canal system. An appropriate and effective method for removing Ca(OH)_2_ is important, and our findings support our recommendation for such a procedure.

## Figures and Tables

**Table 1:** Characteristics of curved root canals

Group	Curvature/°			Radius/mm		
	Mean±s.d.	Maximum	Minimum	Mean±s.d.	Maximum	Minimum
Group 1 (*n*=12)	34.61±7.97	47.7	25.2	7.53±2.27	10.1	3.5
Group 2 (*n*=12)	35.26±7.30	46.2	28.0	7.66±2.01	9.5	4.9
*P*-value	0.465			0.901		

s.d., standard deviations.

**Table 2:** Mean percentage of Ca(OH)_2_ removal with different techniques from simulated root canals in the coronal, middle and apical sections

Group	Group 1 (10% citric acid, *n*=10)				Group 2 (2.5% NaOCl, *n*=10)			
	Whole canal	Cervical 1/3	Middle 1/3	Apical 1/3	Whole canal	Cervical 1/3	Middle 1/3	Apical 1/3
SG1	99.44%±0.46%	100%±0%	100%±0%	96.76%±2.64%	98.89%±0.72%	100%±0%	99.52%±1.00%	94.34%±3.61%
SG2	99.67%±0.29%	100%±0%	100%±0%	98.10%±1.68%	99.37%±0.41%	100%±0%	100%±0%	96.43%±2.33%
SG3	99.79%±0.23%	100%±0%	100%±0%	98.82%±1.34%	99.69%±0.28%	100%±0%	100%±0%	98.25%±1.61%
SG4	99.83%±0.22%	100%±0%	100%±0%	99.02%±1.26%	99.72%±0.26%	100%±0%	100%±0%	98.43%±1.50%

SG1, 30 K file; SG2, F3 instrument; SG3, passive ultrasonic irrigation; SG4, EndoActivator. Values represent mean±standard deviations.

**Table 3:** Mean percentage of Ca(OH)_2_ removal with different techniques from simulated root canals in three sections in the apical 1/3

Group	10% citric acid (*n*=10)			2.5% NaOCl (*n*=10)		
	0–1 mm	1–3 mm	3–5 mm	0–1 mm	1–3 mm	3–5 mm
SG1	87.81%±11.46%	96.48%±4.90%	99.42%±1.28%	73.86%±12.84%	95.30%±5.58%	99.21%±1.36%
SG2	87.85%±9.82%	99.29%±1.50%	100%±0%	79.37%±10.30%	99.21%±1.41%	98.96%±3.28%
SG3	91.22%±9.95%	100%±0%	100%±0%	87.01%±11.95%	100%±0%	100%±0%
SG4	92.73%±9.37%	100%±0%	100%±0%	82.15%±12.75%	100%±0%	100%±0%

SG1, 30 K file; SG2, F3 instrument; SG3, passive ultrasonic irrigation; SG4, EndoActivator. Values represent mean±standard deviations.
